# Identification of key potassium channel genes of temporal lobe epilepsy by bioinformatics analyses and experimental verification

**DOI:** 10.3389/fneur.2023.1175007

**Published:** 2023-07-07

**Authors:** Lin-ming Zhang, Ling Chen, Yi-fei Zhao, Wei-mei Duan, Lian-mei Zhong, Ming-wei Liu

**Affiliations:** ^1^Department of Neurology, The First Affiliated Hospital of Kunming Medical University, Kunming, Yunnan, China; ^2^Yunnan Provincial Clinical Research Center for Neurological Disease, Kunming, Yunnan, China; ^3^Department of Emergency, The First Affiliated Hospital of Kunming Medical University, Kunming, Yunnan, China

**Keywords:** temporal lobe epilepsy, WGCNA, potassium channel genes, target drugs, bioinformatics analyses

## Abstract

One of the most prevalent types of epilepsy is temporal lobe epilepsy (TLE), which has unknown etiological factors and drug resistance. The detailed mechanisms underlying potassium channels in human TLE have not yet been elucidated. Hence, this study aimed to mine potassium channel genes linked to TLE using a bioinformatic approach. The results found that Four key TLE-related potassium channel genes (TERKPCGs) were identified: potassium voltage-gated channel subfamily E member (*KCNA*) 1, *KCNA2*, potassium inwardly rectifying channel, subfamily J, member 11 (*KCNJ11*), and *KCNS1*. A protein–protein interaction (PPI) network was constructed to analyze the relationship between TERKPCGs and other key module genes. The results of gene set enrichment analysis (GSEA) for a single gene indicated that the four TERKPCGs were highly linked to the cation channel, potassium channel, respiratory chain, and oxidative phosphorylation. The mRNA-TF network was established using four mRNAs and 113 predicted transcription factors. A ceRNA network containing seven miRNAs, two mRNAs, and 244 lncRNAs was constructed based on the TERKPCGs. Three common small-molecule drugs (enflurane, promethazine, and miconazole) target *KCNA1, KCNA2*, and *KCNS1*. Ten small-molecule drugs (glimepiride, diazoxide, levosimendan, and thiamylal et al.) were retrieved for *KCNJ11*. Compared to normal mice, the expression of *KCNA1*, *KCNA2*, *KCNJ11*, and *KCNS1* was downregulated in the brain tissue of the epilepsy mouse model at both the transcriptional and translational levels, which was consistent with the trend of human data from the public database. The results indicated that key potassium channel genes linked to TLE were identified based on bioinformatics analysis to investigate the potential significance of potassium channel genes in the development and treatment of TLE.

## Introduction

The second most prevalent neurological disorder is epilepsy, and temporal lobe epilepsy (TLE) is one of the most frequent types in adults (1). TLE comorbidity increases the occurrence of cognitive impairment and behavioral alterations, resulting in low quality of life, career, and finances (2). Although antiepileptic drug treatment, vagus nerve stimulation, and resection of the epileptogenic focus have been applied in TLE, some epilepsy cases still cannot be effectively treated with frequent recurrent seizures (3). Therefore, new strategies are required for early diagnosis and treatment of TLE.

Potassium channels in cell membranes play a critical role in regulating neuronal excitability by controlling the transport of potassium ions across cells (4–6). Several potassium channels are associated with TLE, primarily based on animal experiments (7–10). Research on the association between potassium levels and epilepsy may provide novel insights into the mechanisms and treatments of epilepsy.

However, the mechanisms underlying potassium channels in human TLE have not been elucidated. In this study, we hypothesized that key potassium channel genes are associated with epilepsy pathogenesis. The purpose of this study was to analyze the expression of potassium channels in individuals with TLE from the gene expression omnibus (GEO), and to determine the key potassium channel genes to uncover a new therapeutic target for TLE.

## Methodology

### Data source

The GSE134697 dataset (11), containing two normal neocortex samples from patients without epilepsy and 17 neocortex samples from patients with TLE, was obtained from GEO.^1^ The Molecular Signatures Database (MsigDB)^2^ was used to obtain the file “c5.go.mf.v7.4. symbols.gmt,” and potassium channel-related genes were extracted by referring to the following pathway: GOMF_POTASSIUM_CHANNEL_ACTIVITY, GOMF_POTASSIUM_CHANNEL_INHIBITOR_ACTIVITY and GOMF_POTASSIUM_CHANNEL_REGULATOR_ACTIVITY. Single-cell RNA sequencing (scRNA-seq) data from the GSE140393 dataset were downloaded from the GEO database.

### Weighted gene co-expression network analysis

The transcriptome data of two normal neocortex samples and 17 TLE neocortex samples were analyzed using the R platform’s “WGCNA” (version 1.69) (12). The “Good Samples Genes” function was utilized to carry out clustering of the sample to detect outliers and remove them. To make the co-expression network contented with the distribution of a scale-free network, soft-thresholding power was computed using the pick Soft Threshold function and verified by the correlation between k and p(k) (13). Various modules were identified using the dynamic tree-cutting method, with at least 30 genes in each module (14). Subsequently, to merge similar modules, the threshold was set as 0.2 (15, 16). The associations between these modules the two clinical characteristics were further analyzed. The module with the highest Pearson correlation coefficient was chosen for the subsequent analysis.

### Differentially expressed genes (DEGs) identification

The “limma” in the R package (version 3.44.3) with the voom and empirical Bayes (eBayes) methods was utilized to determine the differentially expressed genes (DEGs) in normal neocortex samples and TLE neocortex samples in the GSE134697 dataset (17). The log_2_-fold changes (log_2_FC), i.e., |log_2_FC| ≥ 0.5 and *p*-value ≤0.05 were deemed as cut-off criteria. A heatmap and a volcano plot were used to display the results.

### Functional enrichment analysis

GO is an important bioinformatics tool for annotating genes and analyzing their products and sequences to understand fundamental biological events such as biological processes (BP), molecular functions (MF), and cellular components (CC). To perform GO analysis, “clusterProfiler” in the R package (version 3. 16.0) was used (18). *p*-value <0.05 was The threshold for enrichment significance was set at *p* < 0.05.

To explore the potential role of TLE related key potassium channel genes (TERKPCGs), Gene Set Enrichment Analysis (GSEA) was performed for a single gene in GSEA (version 2.09). The expression values for each gene were considered as phenotype files, and the correlation coefficients of every gene with other genes were ordered in the gene sets. The “c5.all.v6. 1.symbols.gmt” from MsigDB were selected as the reference gene set. |NES| > 1, NOM value of *p* <0.05, and FDR q-val < 0.25 were set as the thresholds for enrichment significance.

### Construction and analysis of protein–protein interaction network

The Search Tool for the Retrieval of Interacting Genes (STRING) database^3^ was used to build a protein–protein interaction (PPI) network after hiding independent nodes (19). For the construction of the network, nodes with an interaction confidence >0.4 were used. The data were imported into the Cytoscape software (version 3.9. 1) for visual presentation.

### Construction of TF-mRNA and ceRNA regulatory network

To further explore the upstream regulatory molecules of TERKPCGs expression, the upstream transcription factors (TFs) and non-coding RNAs were mined. TFs of the TERKPCGs were predicted using the online website KnockTF.^4^ Target miRNAs of TERKPCGs were predicted using TargetScan and miRDB in the miRWalk database^5^ (20), and it was determined that the miRNAs found in the two databases were the target miRNAs. The StarBase database was used to predict lncRNAs upstream of the miRNAs. Subsequently, lncRNA-miRNA-mRNA and TF-mRNA networks were constructed using the Cytoscape software.

### Identification of phosphorylated sites

Protein modifications and their regulation are associated with protein function. Multiple candidate phosphorylation sites in TERKPCGs have been identified in a post-translational modification database called PhosphoSitePlus^6^ (21).

### Small molecule drugs prediction

To further explore potential drugs that target TERKPCGs and treat TLE, the DrugBank platform,^7^ containing comprehensive molecular data on drugs, their mechanisms, their interactions, and their targets, was used. Small-molecule drugs targeting TERKPCGs were predicted using the DrugBank database (20).

### Animals and *in vivo* study

Male C57 mice were fed and given water as per need and reared at a temperature of 23 C to 25 C, humidity of 45 to 55%, and a 12-h light/dark cycle in a quiet room at Kunming Medical University.

The mice were administered lithium (127 mg/kg) and atropine 18–20 h intraperitoneally (i.p.) before administration of pilocarpine (50 mg/kg, i.p.). Ninety minutes after status epilepticus (SE) establishment, mice were administered diazepam (5 mg/kg, i.p.) to relieve seizures. Behavioral seizures were evaluated according to the Racine limbic seizure categorization, and SE criteria were evaluated when the animal started exhibiting sustained behaviors or higher limbic seizures of score IV or V, with no spontaneous recovery. Similar to the experimental mice, the control mice were injected with NaCl (0.9%) rather than pilocarpine. Following acute exacerbation, the mice were administered 5% glucose and sodium chloride (5 mL, once daily, i.p.) during the incubation period. Upon entering the chronic phase (after 28 days), the mice were sacrificed by cervical dislocation and anesthetized with 1% pentobarbital sodium (50 mg/kg body mass; Wuhan Dinghui Chemical Co., Ltd., Wuhan, China). Brain tissues were collected from six mice in each group to determine the expression levels of TERKPCGs. This protocol was approved by the Committee of Ethics of Animal Experiments at Kunming Medical University (Permit Number: SCXK 2011-0015). Eight mice showed spontaneous seizures in the chronic phase and their behavior was monitored using a camera. Brain samples were collected from mice according to Racine limbic seizure categorization. Two mice died and were thus excluded from the treatment group.

### Extraction of RNA and quantitative real-time polymerase chain reaction

Total RNA was extracted from six normal mouse brain tissues and six epileptic mouse brain tissues using Nuclezol LS RNA Isolation Reagent according to the manufacturer’s guidelines (ABP Biosciences Inc.). As per the given protocol, total RNA was converted into cDNA through reverse transcription employing the SureScript-First-strand-cDNA-synthesis-kit (GeneCopoeia). qPCR was then implemented using BlazeTaq™ SYBR^®^ Green qPCR Mix 2.0 (GeneCopoeia). The thermocycling program employed for qPCR was as follows: one cycle of initial denaturation for 30 s at 95°C, followed by 40 cycles of denaturation for 10 s at 95°C, annealing for 20 s at 60°C, and extension for 30 s at 72°C. The primer sequences used are listed in Table 1. The corresponding expression levels were normalized to the endogenous control GAPDH and calculated using the 2^−ΔΔCq^ method (22). The differences between the two groups were compared using the Student’s *t*-test. In the two-tailed statistical analysis, a *p*-value <0.05 was deemed statistically significant.

**Table 1:** Primer sequences for quantitative real-time polymerase chain reaction (qRT-PCR).

Genes	Forword	Reverse
KCNA1	GCTAGTATGAGGGAGTTAGGG	TCGGTGGTAGAAATAGTTGAA
KCNA2	CCCCAAGAAACGGATGAGGTA	GAAGAAAGGGTCGGTGAAGGA
KCNJ11	CTTGGAAGGCGTGGTAGAAAC	CGTCAGCTAGGTAGGAGGTGC
KCNS1	CCCATCACCATCATCTTCAAC	AGAGACACATCCGATACCCCG
GAPDH	CCTTCCGTGTTCCTACCCC	GCCCAAGATGCCCTTCAGT

### Immunohistochemistry

Hippocampal specimens from mice were polyformaldehyde-fixed, immersed in paraffin, and sliced into 4 μm sections. The sections were dewaxed, rehydrated with xylene, and washed with alcohol and PBS gradient. After heating in citric acid buffer (pH 6.0) for 15 min, antigens were retrieved. TBS/H2O2 was used to block endogenous peroxidase activity. After incubation with primary antibodies for 1 h at room temperature, the sections were incubated with secondary antibodies for 30 min at 37°C. Primary antibodies against mouse monoclonal *KCNA1* (bs-8691R), *KCNA2* (bs- 12182R), *KCNJ11* (bs-2436R), and *KCNS1* (bs- 16916R) (1:1000) were purchased from BIOSS (Beijing, China). Rabbit anti-mouse IgG H&L (HRP) (bs-0296R) incubation kit was purchased from ZSGB-Bio (Beijing, China). The antibodies were used according to the manufacturer‟s guidelines. Immunohistochemical staining was performed by exposing the sections to diaminobenzidine (DAB) as a chromogen. The sections were counterstained with hematoxylin, dehydrated in ethanol, and clarified in xylene. Observations and photographs were obtained using a microscope (Zeiss, Lab. A1). According to a previously described method (23), after staining, five high-power fields (×200) were randomly selected on each slide, and the average proportion of positive expression in each field was counted using the true color multi-functional cell image analysis management system (Image-Pro Plus; Media Cybernetics, Rockville, MD, USA).

### Statistics analysis

Differences in TF expression between groups were analyzed using the Wilcoxon test. Student’s *t*-test was conducted for differences in the expression of key TERKPCGs in clinical mouse brain tissues and hippocampal specimens. Statistical analysis was employed with *p* < 0.05 as a significant difference.

## Results

### Identification of the key module genes for TLE

**Figure 1:**
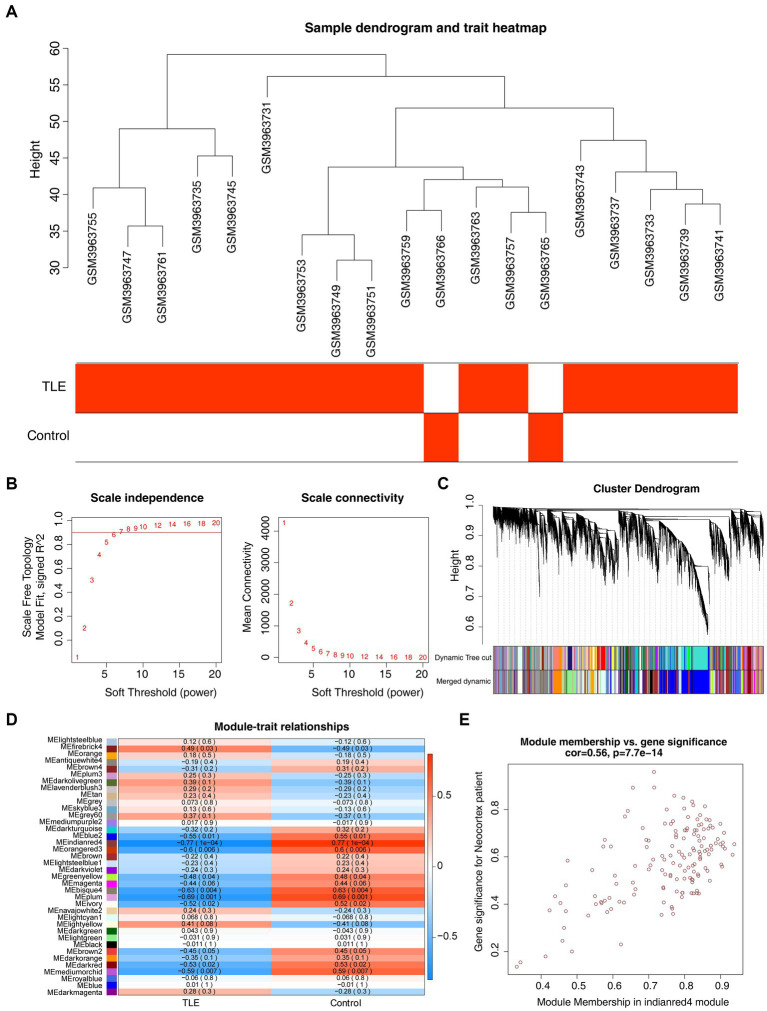
Gene co-expression network analysis (WGCNA) and key modules identification. **(A)** Sample clustering dendrogram and trait heatmap of cohorts in GSE134697 to remove outliers. **(B)** Analysis of the scale-free fit index (left) and the mean connectivity (right) for various soft-thresholding powers. **(C)** Clustering dendrogram of all genes based on similar expression patterns, with assigned module colors. **(D)** The correlation between different modules and clinical traits. **(E)** Scatterplot of Gene Significance (GS) and Module Membership (MM) in MEindianred4.

To identify the key modules highly linked to TLE, we structured WGCNA on the genes in the GSE134697 dataset. No obvious outlier samples were removed through sample clustering, and *β* = 6 (scale-free R^2^ = 0.9) was selected to develop a scale-free network (Figures 1A,B). Based on the similar expression patterns of all genes in the cohorts, average dynamic tree cutting and hierarchical clustering were conducted, in which the retrieved modules were merged to a minimum size of 30. Following eigengene calculation, 38 modules were clustered and merged (Figure 1C). Subsequently, the association between each module and two clinical characteristics (TLE and healthy controls) was assessed, suggesting that MEindianred4 (|cor| = 0.77, *p* = 0.0001) showed the strongest association with TLE, and the close correlation of gene significance and module significance was displayed with cor = 0.56 (*p* < 0.0001) (Figures 1D,E). Thus, 151 genes from this module were used for further analyses (Supplementary Table S1).

### Identifying and exploration of DEGs in TLE

**Figure 2:**
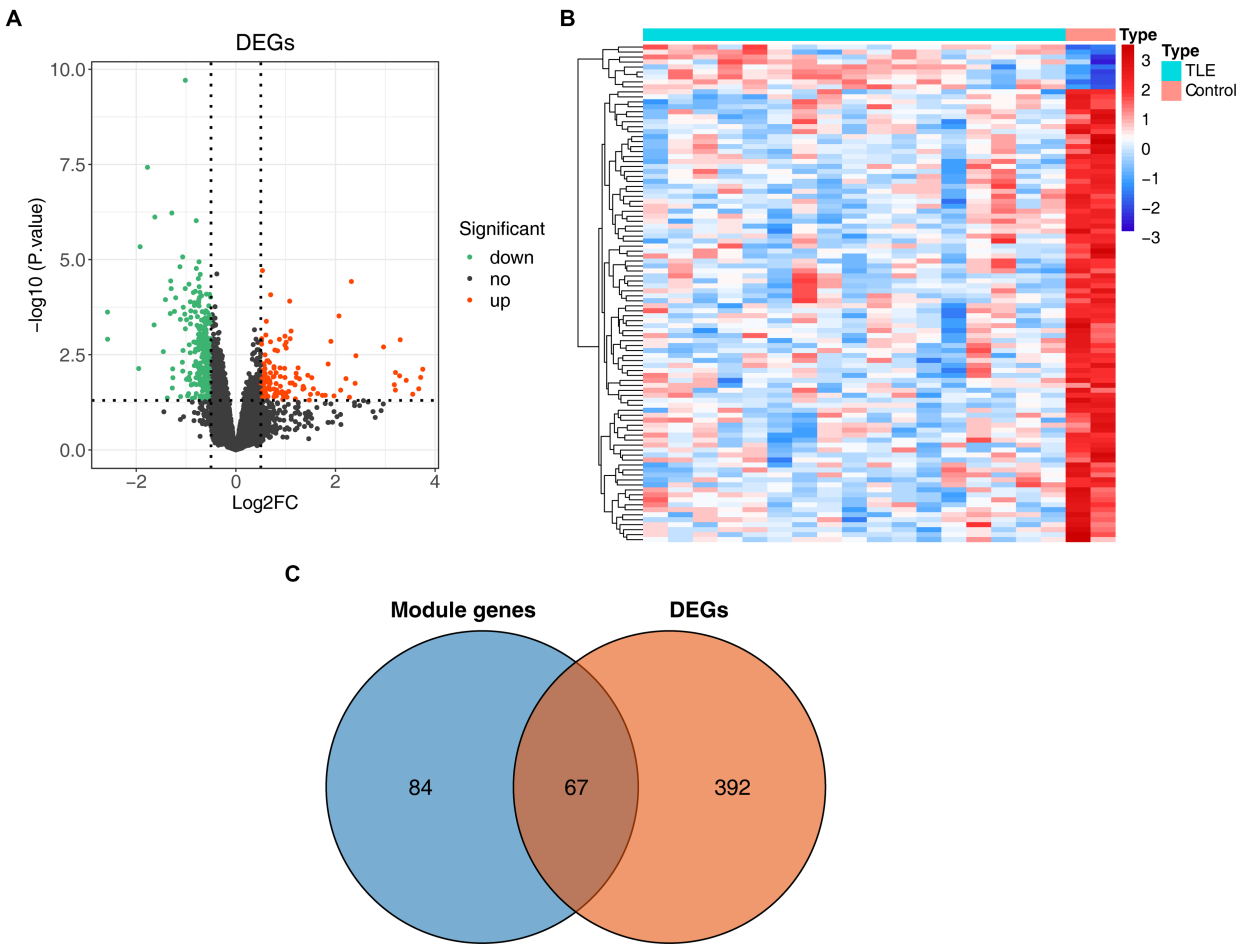
Identification of differentially expressed genes (DEGs) in TLE. **(A)** The volcano map of DEGs between normal and TLE samples. **(B)** The heatmap of top100 DEGs. **(C)** Venn diagram for TLE-related DEGs.

We further performed differential expression analysis of gene expression data from the GSE134697 dataset. Based on the established criteria, 459 DEGs (containing 144 upregulated and 315 downregulated genes) were identified between the normal and TLE samples (Figures 2A,B; Supplementary Table S2). Subsequently, 67 common genes from 151 key module genes and these DEGs were selected, namely, TLE-related DEGs (Figure 2C; Supplementary Table S3). As shown in Supplementary Table S4, in the CC ontology, the TLE-related DEGs were significantly associated with the ion channel complex, transmembrane transporter complex, and potassium channel complex. Concerning MF, these genes were related to metal ion transmembrane transporter activity, ion channel activity, and voltage-gated potassium channel activity.

### Identification of TERKPCGs and gene set enrichment analysis

**Figure 3:**
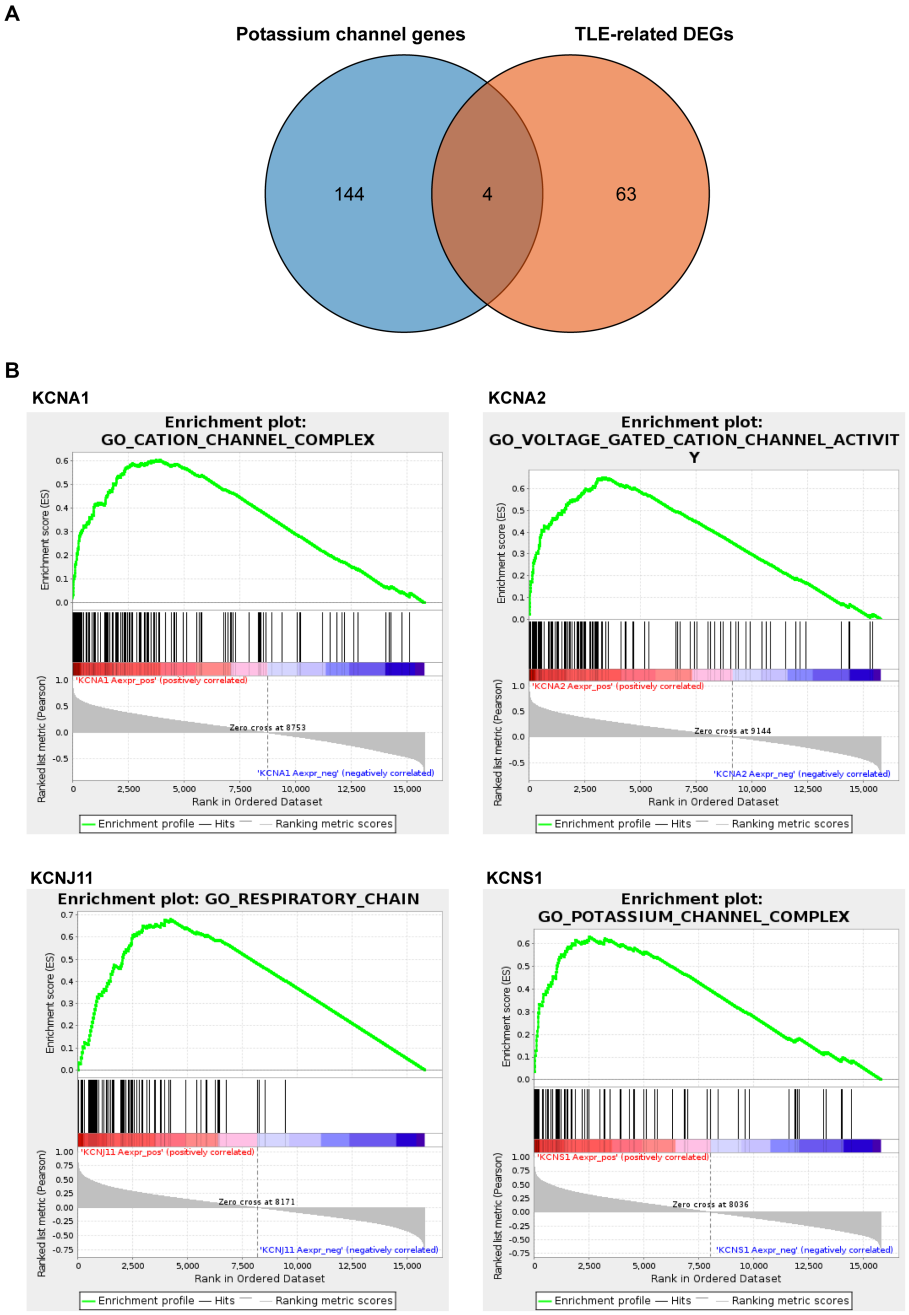
Identification and analysis of TLE-related key potassium channel genes (TERKPCGs). **(A)** Venn diagram for TERKPCGs. **(B)** Single-gene Gene set enrichment analysis (GSEA) of four TERKPCGs. (Top) Enrichment score (ES) line chart: the display of the ES value as it is calculated to each position when the analysis is sorted along the ranking list. The score at the highest peak (the farthest vertical distance) is the ES value of the gene set. (Middle) Lines are used to mark the position of members from the gene set in the gene sorting list, and the black line represents that the genes from the sorting gene table exist in the functional annotation gene set of the current analysis. Leading edge subset is the corresponding gene from (0,0) to the highest peak ES. (Bottom) After sorting, the rank values of all genes were distributed. The genes corresponding to the red part of the heat map were highly positively correlated to the TERKPCG, and the genes corresponding to the blue part were highly negatively correlated to the TERKPCG. The pearson correlation coefficient of each gene was displayed in a gray area map.

Next, we downloaded 148 potassium channel-related genes from MsigDB (Supplementary Table S5) and selected the intersection with 67 TLE-related DEGs to obtain the TLE-related key potassium channel genes, where *KCNA1*, *KCNA2*, *KCNJ11*, and *KCNS1* were selected as TERKPCGs (Figure 3A). A PPI network was constructed to analyze the interrelationship between TERKPCGs and other key module genes (Supplementary Figure S1).

Furthermore, GSEA for a single gene in the expression data of the GSE134697 dataset based on Gene Ontology gene sets was conducted to evaluate the potential mechanism of TERKPCGs in TLE. The detailed enrichment results of GO terms for each TERKPCG are shown in Supplementary Table S6, and the top 10 were positively correlated with each TERKPCG, as displayed in Table 2. We noted that the functional term mostly correlating with *KCNA1* was the cation channel complex, and voltage-gated cation channel activity was mostly linked to *KCNA2*. Meanwhile, the respiratory chain was most related to *KCNJ11*, and the top functional term relevant to *KCNS1* was the potassium channel complex (Figure 3B).

**Table 2:** The top10 enrichment results of each TLE-related key potassium channel gene (TERKPCG) by Gene set enrichment analysis (GSEA).

KCNA1					
NAME	SIZE	ES	NES	NOM p-val	FDR q-val
GO_CATION_CHANNEL_COMPLEX	139	0.6039766	2.8596067	0	0
GO_POTASSIUM_CHANNEL_COMPLEX	80	0.6464319	2.8221097	0	0
GO_SYNAPTIC_SIGNALING	361	0.5191881	2.7956884	0	0
GO_DELAYED_RECTIFIER_POTASSIUM_CHANNEL_ACTIVITY	30	0.76757	2.7920687	0	0
GO_VOLTAGE_GATED_ION_CHANNEL_ACTIVITY	155	0.5769672	2.788813	0	0
GO_VOLTAGE_GATED_POTASSIUM_CHANNEL_ACTIVITY	73	0.6447623	2.7679226	0	0
GO_REGULATION_OF_SYNAPSE_STRUCTURE_OR_ACTIVITY	208	0.5488174	2.7588754	0	0
GO_TRANSPORTER_COMPLEX	261	0.527908	2.7167165	0	0
GO_VOLTAGE_GATED_CATION_CHANNEL_ACTIVITY	112	0.5808316	2.6590855	0	0
GO_EXOCYTIC_VESICLE_MEMBRANE	54	0.6517453	2.646571	0	0
KCNA2					
NAME	SIZE	ES	NES	NOM p-val	FDR q-val
GO_VOLTAGE_GATED_CATION_CHANNEL_ACTIVITY	112	0.6513996	2.8166625	0	0
GO_VOLTAGE_GATED_ION_CHANNEL_ACTIVITY	155	0.593186	2.7048934	0	0
GO_POTASSIUM_CHANNEL_COMPLEX	80	0.6397141	2.6886604	0	0
GO_VOLTAGE_GATED_POTASSIUM_CHANNEL_ACTIVITY	73	0.657539	2.6878352	0	0
GO_CATION_CHANNEL_COMPLEX	139	0.6045695	2.6735845	0	0
GO_CATION_CHANNEL_ACTIVITY	226	0.5566384	2.619445	0	0
GO_GATED_CHANNEL_ACTIVITY	256	0.5450291	2.593696	0	0
GO_TRANSPORTER_COMPLEX	261	0.5313092	2.5284357	0	0
GO_POTASSIUM_CHANNEL_ACTIVITY	98	0.581334	2.4802358	0	0
GO_POTASSIUM_ION_TRANSPORT	124	0.5585991	2.451978	0	0
KCNJ11					
NAME	SIZE	ES	NES	NOM p-val	FDR q-val
GO_RESPIRATORY_CHAIN	73	0.6802167	2.8653443	0	0
GO_OXIDATIVE_PHOSPHORYLATION	75	0.663591	2.8199456	0	0
GO_INNER_MITOCHONDRIAL_MEMBRANE_PROTEIN_COMPLEX	75	0.6366258	2.789315	0	0
GO_ELECTRON_TRANSPORT_CHAIN	87	0.6318213	2.7407804	0	0
GO_MITOCHONDRIAL_MEMBRANE_PART	129	0.5728427	2.6561408	0	0
GO_MITOCHONDRIAL_PROTEIN_COMPLEX	100	0.5947027	2.6416457	0	0
GO_CELLULAR_RESPIRATION	132	0.5559288	2.6052012	0	0
GO_MITOCHONDRIAL_RESPIRATORY_CHAIN_COMPLEX_ASSEMBLY	63	0.6404683	2.6039183	0	0
GO_NADH_DEHYDROGENASE_COMPLEX	42	0.6808007	2.5775912	0	0
GO_VOLTAGE_GATED_POTASSIUM_CHANNEL_ACTIVITY	73	0.6060878	2.5591702	0	0

KCNS1					
NAME	SIZE	ES	NES	NOM p-val	FDR q-val
GO_POTASSIUM_CHANNEL_COMPLEX	80	0.6302192	2.783882	0	0
GO_RESPIRATORY_CHAIN	73	0.6240602	2.7153847	0	0
GO_VOLTAGE_GATED_POTASSIUM_CHANNEL_ACTIVITY	73	0.6039534	2.6524305	0	0
GO_CATION_CHANNEL_COMPLEX	139	0.5449558	2.6484447	0	0
GO_ELECTRON_TRANSPORT_CHAIN	87	0.5856932	2.6234436	0	0
GO_OXIDATIVE_PHOSPHORYLATION	75	0.608048	2.6176531	0	0
GO_MITOCHONDRIAL_MEMBRANE_PART	129	0.5411719	2.6165476	0	0
GO_INNER_MITOCHONDRIAL_MEMBRANE_PROTEIN_COMPLEX	75	0.5924099	2.605312	0	0
GO_DELAYED_RECTIFIER_POTASSIUM_CHANNEL_ACTIVITY	30	0.711552	2.5940406	0	0
GO_SYNAPTIC_SIGNALING	361	0.4685044	2.5921729	0	0

SIZE, the number of genes enriched in GO entry; ES, Enrichment score; NES, Normalized corrected ES values; NOM p-val, Statistical significant value of ES; FDR q-val, The probability of false positive results, is the corrected *p* value by multiple hypothesis testing.

### The underlying regulatory mechanism targeting four TERKPCGs

**Figure 4:**
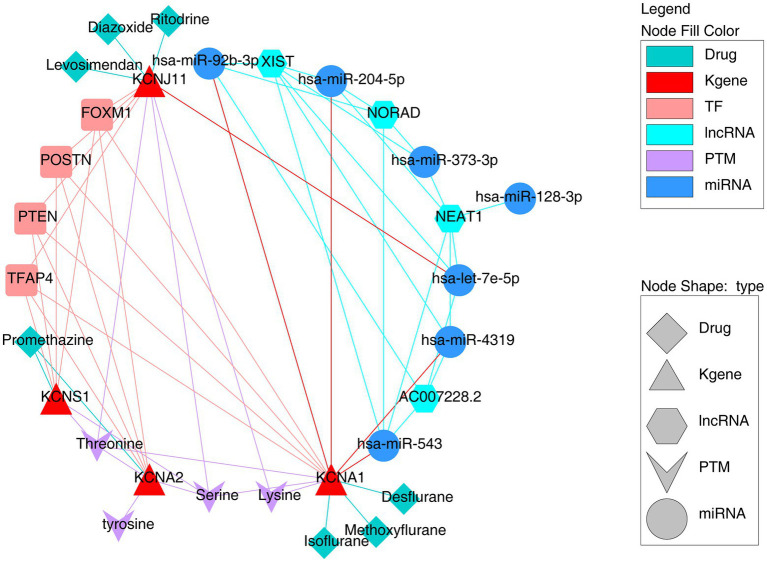
The comprehensive regulation network targeting four key TERKPCGs.

To further investigate the underlying regulatory network targeting key TERKPCGs, the potential TFs binding sites and correlated miRNA targets were predicted using the KnockTF and miRWalk tools. As shown in Supplementary Figure S2, a TF mRNA network was established using four TERKPCGs and 113 predicted transcription factors. Interestingly, these TERKPCGs were commonly regulated by four transcription factors (*TFAP4*, *FOXM1*, *POSTN*, *and PTEN*), although their expression levels were not significantly different between the case and control groups (Supplementary Figure S3). On the other hand, six miRNAs (*hsa-miR-204-5p*, *hsa-miR-128-3p*, *hsa-miR-373-3p*, *hsa-miR-92b-3p*, *hsa-miR-54*3, and *hsa-miR-4,319*) targeting *KCNA1* and one miRNA, *hsa-let- 7e-5p* targeting *KCNJ11* were predicted, and corresponding lncRNAs upstream of which were shown in Supplementary Figure S4, including the regulatory axes LINC02242-hsa-let-7e-5p-*KCNJ11* and LINC01963-hsa-miR-204-5p-*KCNA1*. Meanwhile, the TOP4 lncRNAs common to most miRNAs are exhibited in Figure 4, for example, NEAT1, XIST, AC00728.2, and NORAD, providing new information for studying the role of TF regulation and ceRNA regulatory axes in TLE development.

Moreover, considering the influential role of protein post-translational modifications (PTMs) and potassium channels in the nervous system (24, 25), an in-depth study of PTMs is essential for elucidating the functional interactions of the key TERKPCGs in the pathogenesis of TLE. The PhosphoSitePlus online website was employed to identify the phosphorylation and ubiquitination modifications of the four TERKPCGs. Detailed modification loci for each protein are presented in Table 3. Phosphorylation modifications of serine, threonine, tyrosine, and lysine were mainly relevant to the four TERKPCGs (Figure 4).

**Table 3:** Histone modification sites of proteins encoded by each TERKPCG.

Gene Symbols	Modified site	Sequence	Amino site	Amino	Modified type
KCNA1	S445-p	DSDLSRRssStMSKS	Ser445	Serine	Phosphorylation
	S446-p	SDLSRRssStMSKSE	Ser446	Serine	Phosphorylation
	T448-p	LSRRssStMSKSEYM	Thr448	Threonine	Phosphorylation
	K49-ub	LRFETQLkTLAQFPE	Lys49	Lysine	Ubiquitylation
KCNA2	Y132-p	MFREDEGyIKEEERP	Tyr132	Tyrosine	Phosphorylation
	Y415-p	VIVSNFNyFyHRETE	Tyr415	Tyrosine	Phosphorylation
	Y417-p	VSNFNyFyHRETEGE	Tyr417	Tyrosine	Phosphorylation
	S440-p	TSCPKIPssPDLKKS	Ser440	Serine	Phosphorylation
	S441-p	SCPKIPssPDLKKSR	Ser441	Serine	Phosphorylation
	S451-p	LKKSRSAstISKSDY	Ser451	Serine	Phosphorylation
	T452-p	KKSRSAstISKSDYM	Thr452	Threonine	Phosphorylation
KCNJ11	T180-p	QAHRRAEtLIFSKHA	Thr180	Threonine	Phosphorylation
	S208-p	RVGDLRKsMIISATI	Ser208	Serine	Phosphorylation
	T224-p	MQVVRKTtSPEGEVV	Thr224	Threonine	Phosphorylation
	K332-ub	RYSVDYSkFGNTIKV	Lys332	Lysine	Ubiquitylation
	S372-p	RGPLRKRsVPMAKAK	Ser372	Serine	Phosphorylation
KCNS1	S28-p	PTPLGGRstETFVSE	Ser28	Serine	Phosphorylation
	T29-p	TPLGGRstETFVSEF	Thr29	Threonine	Phosphorylation
	T503-p	SLETSREtsQEGQsA	Thr503	Threonine	Phosphorylation
	S504-p	LETSREtsQEGQsAD	Ser504	Serine	Phosphorylation
	S509-p	EtsQEGQsADLESQA	Ser509	Serine	Phosphorylation

### Drugs prediction

To discover potential drugs that can function in TLE, we identified potential drugs targeting the proteins encoded by TERKPCGs, as shown in Supplementary Table S7. Considering the downregulation patterns of four TERKPCGs, seven drugs with activator actions were selected and imaged (Figure 4), where promethazine might reverse the expression of *KCNA2*, *KCNS1*, and diazoxide, levosimendan, and ritodrine were considered as inducers of *KCNJ11.* Among isoflurane, methoxyflurane and desflurane target *KCNA1*. However, further animal studies and clinical validation are required for clinical application.

### The expression of TERKPCGs in the mouse model with TLE

**Figure 5:**
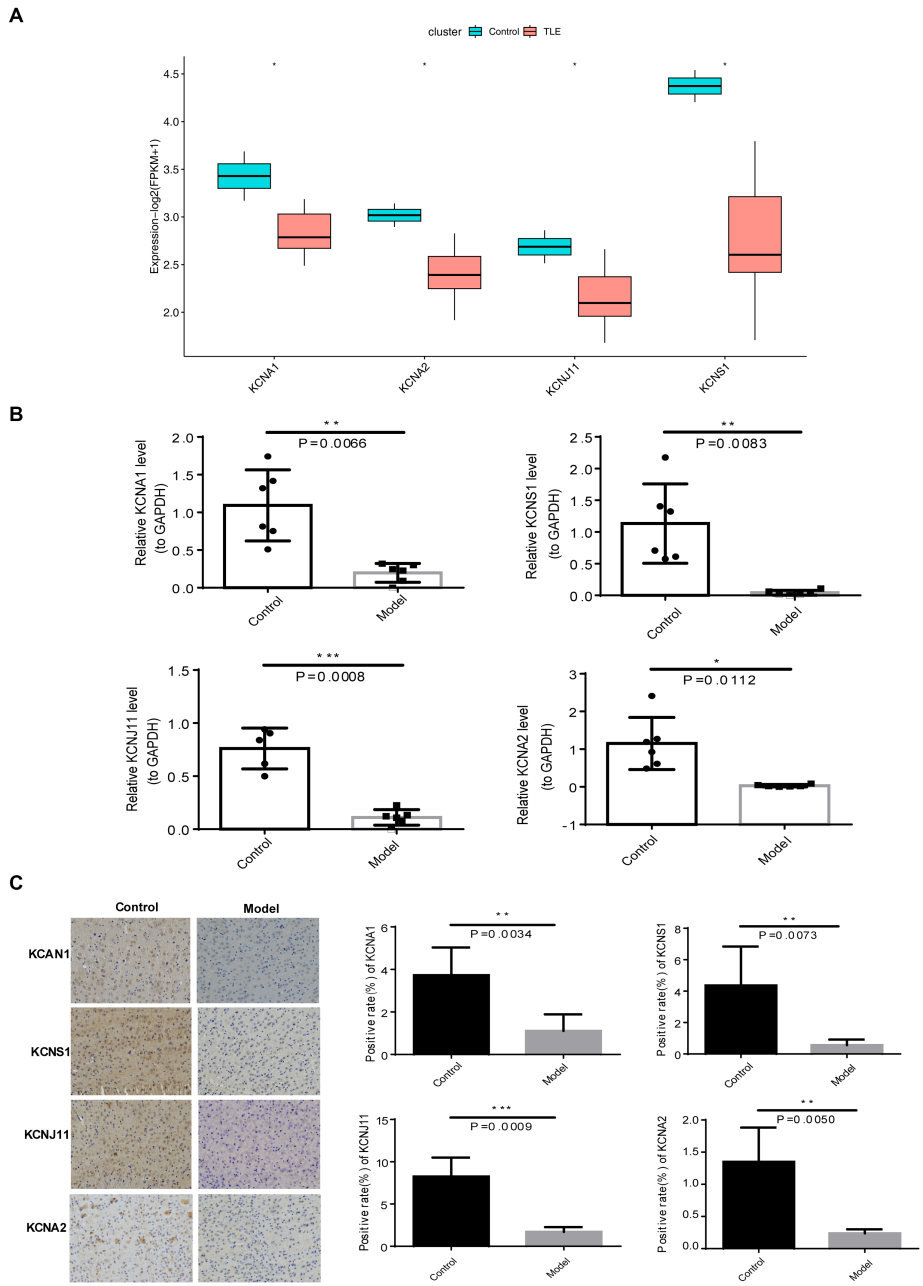
Expression verification of four TERKPCGs. **(A)** Boxplot for the expressions of four TERKPCGs in GSE134697. **(B)** The relative mRNA expression level of four TERKPCGs in brain tissue of control and epilepsy model mice. **(C)** Protein expression levels of TERKPCGs in brain tissue of control and epilepsy model mice, including the IHC images and the corresponding positive expression of IHC images. *p < 0.05, **p < 0.01, ***p < 0.001.

Eventually, the expression patterns of four TERKPCGs identified above was exhibited in Figure 5A, where among the expressions of *KCNA1*, *KCNA2*, *KCNJ11*, and *KCNS1* were decreased in TLE cohorts of GSE134697. Moreover, the expression of the four TERKPCGs at the transcriptional and translational levels was further confirmed using quantitative real-time polymerase chain reaction (qRT-PCR) and IHC analyses of clinical samples. We first established a mouse model of epilepsy and simultaneously established a control group. Brain tissues from six control mice and six epilepsy mouse models were used for the experiments. As shown in Figure 5B, *KCNA1*, *KCNA2*, *KCNJ11*, and *KCNS1* were all downregulated at the mRNA level in the brain tissue of epileptic mice compared to control mice. IHC results indicated downregulation of the expression of the four genes at the protein level in the brain tissue of epileptic mice. This is also illustrated by the statistics on the positive rate of IHC in the group of six mice (Figure 5C). The expression profiles of *KCNA1*, *KCNA2*, *KCNJ11*, and *KCNS1* in mice were consistent with the results of the human data from the GSE134697 dataset.

## Discussion

Potassium channels exiting nerve cell membranes play a critical role in regulating neuronal excitability by controlling the transport of potassium ions across cells. Changes in potassium channel gene expression play an important role in the occurrence, development, treatment, and prevention of epilepsy. To date, there is a lack of studies on potassium channel genes in epilepsy. Therefore, this study aimed to probe potassium channel genes linked to TLE using a bioinformatic approach. The results indicated that key potassium channel genes linked to TLE were identified based on bioinformatics analysis to investigate the potential significance of potassium channel genes in the development and treatment of TLE.

In this study, 151 key module genes identified using WGCNA were significantly associated with TLE. A total of 459 differentially expressed genes (DEGs) were screened in TLE samples and compared with the controls. In total, 67 genes were obtained from the intersections of 151 key genes, and DEGs were considered crucial genes linked to TLE. GO functional annotations and KEGG pathway analyses of the crucial 67 genes linked to TLE were performed to uncover the mechanisms of TLE. The findings indicated that These genes mainly participated in many GO terms, including ion channel complexes, transporter complexes, transmembrane transporter complexes, and main axons. However, these crucial genes were not involved in KEGG pathway-related regulation.

Previous research has shown that three of the four key potassium channel genes (*KCNA1*, *KCNA2*, *KCNJ11*, and *KCNS1*) play vital roles in seizures and epilepsy (8–10). Four key potassium channel genes were screened based on the intersection of crucial 67 genes and genes associated with potassium channels from the MsigDB database. Based on previous studies (26–29), RT-PCR and immunohistochemistry analyses of the expression levels of four key genes in the brains of TLE mice validated the results obtained from bioinformatic analyses, showing that mRNA and protein expression of these four key genes were remarkably decreased in TLE mice compared to the control. The low expression levels of these four genes may be associated with the progression of epilepsy. An imbalance between inhibitory and excitatory neurons plays an important role in epilepsy (30, 31). Potassium channel downregulation might impair excitatory neuron repolarization and decrease the threshold for action potential (AP) firing, resulting in network hyperexcitability and increased seizure susceptibility (32).

*KCNA1* encodes the alpha subunit of KV1. 1, which belongs to the Shaker subfamily (Kv1). KV1. 1 mediating the outflow of K+ regulates action potential propagation and shape, neuronal repetitive firing properties, membrane repolarization, and neurotransmitter release, which are positioned in the axons and axon terminals of the hippocampal trisynaptic circuit (33–35). Mutations in *KCNA1* are associated with multiple epilepsies (36, 37). Studies have shown that *KCNA1* targeted deletions result in TLE and sudden death in animal models of epilepsy (35–45). The *KCNA1* gene encodes the alpha subunit of the Kv1. 1 channel (a delayed rectifier, voltage-gated potassium channel) in humans and mice, and its functional mutations are linked with partial epilepsy, episodic ataxia, sleep disorders, and sudden death in epilepsy (26, 38, 41–44, 46–49). In Kv1. 1 knockout (KO) mice, infrared telemetric actimetry was performed immediately after weaning and continued until the animals suddenly died. *KCNA1*-null mice with a targeted deletion of the Kv1. 1 potassium channel α subunit protein have severe spontaneous recurrent seizures (SRS) and are frequently used as epilepsy mouse models (33–39). Overexpression of Kv1. 1 can reduce the frequency of brief (1 s), and high-frequency epileptiform discharges (49). Therefore, upregulation of *KCNA1* can successfully suppress seizures in a rodent model of intractable TLE (50, 51) and offers a potential new treatment option for TLE in humans. The finding in the current study that *KCNA1* was downregulated in clinical case samples is consistent with previous literature, providing a theoretical basis for subsequent pathway analysis.

*KCNA2*, also a member of the Shaker subfamily (Kv1), encodes Kv1.2. Kv1.2 channels are widely expressed voltage-dependent potassium channels all over the central and peripheral nervous systems located within Kv1. 1 along the axons and axon terminals, as well as at presynaptic sites (52, 53). High-density clustering of Kv1. 1 and Kv1.2 is located in the juxta paranodal area neighboring the nodes of Ranvier of mammalian axons, at axonal initial segments, in the pinceau area of cerebellar basket cells, in the hippocampus, cortex, and auditory brainstem (44, 54). Kv1.2 are low-voltage activated gradually inactivating channels that open in response to small depolarizations near the resting potential (42, 55). They are involved in the initiation and shaping of action potentials, influencing the firing patterns of the action potential, controlling the excitability of neurons, and neurotransmitter release (56). Downregulation of Kv1.2 which likely increases seizure susceptibility, impairs excitatory neuron repolarization and decreases the threshold for AP firing, activating network hyperexcitability (8). Echoing the dysregulation results for Kv1.2, the mRNA and protein levels of *KCNA2* were downregulated in the clinical TLE samples. In addition, Imbrici found that the E236K variant in *KCNA2* exhibited voltage-dependent activation, shifting toward negative potentials and slower kinetics of deactivation and activation, causing early onset of epilepsy (32). Uysal reported that patients with mutations in *KCNA2* showed early onset developmental and epileptic encephalopathies (57). *KCNJ11* plays a key role in controlling K+ channel selectivity (57). More than 130 mutations have been reported in *KCNJ11*, which cause psychomotor developmental delay and epilepsy during the infantile period with the most severe form (58). However, the involvement of *KCNJ11* and *KCNS1* in epilepsy has not been reported thus far, and it was first found that the mRNA and protein levels of these two genes were downregulated in TLE samples compared to normal controls, which remains to be confirmed by further studies.

Gene set enrichment analysis (GSEA) revealed that the functions of *KCNA1* and *KCNA2* were enriched in signal transduction, including cation channel activity, potassium ion channel activity, and ion voltage gating. *KCNJ11* functions in the respiratory chain, oxidative phosphorylation, mitochondrial intima protein complex, potassium channel complex, cation channel, and charge transfer. *KCNS1* is primarily involved in the potassium channel complex and respiratory chain.

In this study, the phosphorylation modification of serine, threonine, tyrosine, and lysine was mainly relevant to the four TERKPCGs. The study found that Targeted post translational modifications of drugs have the potential to restore tetratricopeptide repeat-containing Rab8b-interacting protein phosphorylation to reduce TLE excitability (59). D-serine mitigates cell loss associated with temporal lobe epilepsy (60). Serine/threonine protein kinases are targets of rapamycin (mTOR), and mTOR activation induces the expression of voltage-gated potassium channel Kv1. 1 in TLE animal models, indirectly reflecting the correlation between TERKPCGs and serine/threonine phosphorylation (61). Transfusion of genetically modified tyrosine kinase receptor B into the hippocampus increases the susceptibility or severity of seizures (62). Controlling the expression of the silk/threonine kinase With-no-lysine kinase 3 may be a new therapeutic target for epilepsy (63). The above research on amino acid kinases provides a theoretical basis for the correlation between key TERKPCGs and amino acid phosphorylation in our study, and is expected to target key genes to further explore their case mechanisms in TLE.

To further investigate the mechanism of the transcriptional regulation of these four genes, we constructed TF mRNA and ceRNA networks. In the TF mRNA network, we found four identical transcription factors, including TFAP4, FOXM1, POSTN, and PTEN, which may regulate the transcription of the four genes. In Dravet syndrome-iPSC GABA lines, there was a remarkable upregulation of FOXM, which is a positive regulator of disrupted pathways for histone modification and cell cycle regulation (64). Glutamatergic density and strength of glutamatergic cells by PTEN. Loss of PTEN can significantly enhance the formation of excitatory synapses in neurons, making them hyperexcitable (65). POSTN promotes neural stem cell proliferation and differentiation following hypoxic–ischemic injury (66). Clarithromycin ameliorates early brain injury after subarachnoid hemorrhage via suppressing postn-related pathways in mice (67). TFAP4 focuses on various cancers, but no research has been conducted on brain injury or epilepsy (68, 69). In this study, the expression of four key TFs between the disease and control groups was not significantly different, indicating that changes in the expression of these transcription factors may not be the key factors leading to changes in the expression and mechanism of key TERKPCGs in TLE patients. Considering the small sample size of the dataset, this discovery needs to be further validated by collecting more clinical samples in the future. In the ceRNA network, possible ceRNA regulatory mechanisms of the two potassium channel genes were revealed, providing a basis for future research on specific mechanisms. Previous studies have found that microRNA-204 inhibits epileptic discharges by regulating TrkB-ERK1/2-CRB signals in cultured hippocampal neurons (66). MiR-204-5p is involved in the regulation of hippocampal sclerosis and focal cortical dysplasia, and participates in the occurrence and development of epilepsy (70). miRNA let-7e regulates the expression of caspase-3 during apoptosis in PC12 cells following anoxia/reoxygenation injury (71). TLE alters the expression levels of miRNA let-7e in the hippocampus, and deregulated miRNA let-7e may be involved in the pathogenesis of epilepsy directly or indirectly (72). Therefore, LINC02242-hsa-let-7e-5p-*KCNJ11* and LINC01963-hsa-miR-204-5p-*KCNA1* might be involved in the regulation of epilepsy and brain injury. However, the regulatory relationships between these factors require further exploration. In this study, the TOP4 lncRNAs common to most miRNAs were exhibited, the studies have found that The NEAT1/NORAD/XIST has-miR-204 axis regulates the development of Kawasaki disease (KD) (73). Further research has found that these three key lncRNAs together act as potential genes in interstitial lung disease (SSC ILD) and breast cancer in systemic sclerosis (74, 75). Therefore, we aimed to target the interactions between these three key genes to explore their biological significance in TLE.

The DrugBank database was used to identify the target drugs of the four key potassium channel genes to explore potential anti-epilepsy drugs. Molecular docking results showed that enflurane, promethazine, and miconazole targeted *KCNA1*, *KCNA2*, and *KCNS1*. Studies have found that enflurane has anticonvulsant effects in cat epilepsy models (76). There is little exacerbation of preexisting epileptic foci by enflurane, the only exception being the case of certain myoclonic-type epilepsies, such as progressive myoclonic epilepsy and photosensitive epilepsy (76). Promethazine reduces brain injury after ischemic stroke through PKC-δ/NOX/MnSOD and RIP1-RIP3 regulated activation of the NLRP3 inflammasome following ischemic stroke (77, 78). Miconazole exerts disease-modifying effects in epilepsy by suppressing neuroinflammation via the NF-κB pathway and iNOS production (79). The application of isoflurane as an anesthetic in TLE research has been reported for a long time (80). Volatile anesthetics such as isoflurane and desflurane have inhibitory effects on human Kv channels at various clinical concentrations (81). Our study suggests that their inhibitory effects on TLE may target KCNA1. Other drugs have not been further studied in TLE, and further confirmation is required.

However, this study has some limitations. First, the sample size used in this study was small, although supplemented with mouse models for testing, a larger sample of TLE patients was collected for further analysis. Second, the expression of key genes in neuronal cells has not been detected in the MsigDB database, which is inconsistent with *in vitro* experiments and needs to be confirmed by further research in the future. Thirdly, the binding sites among ceRNAs targeting key TERKPCGs were predicted and need to be examined using more functional experiments. Moreover, the clinical use of inducers of these TERKPCGs remains to be tested in TLE progression in the future.

## Conclusion

We identified four key potassium channel genes, *KCNA1*, *KCNA2*, *KCNJ11*, and *KCNS1*, that are associated with the pathogenesis of epilepsy. Because our aim was to screen key genes related to potassium channel disorders, the four genes obtained may indirectly affect the balance between inhibitory and excitatory neurons, mainly through the relevant mechanism of potassium channel downregulation. Therapeutic strategies targeting these genes will be our future research direction.

## Data availability statement

The datasets presented in this study can be found in online repositories. The names of the repository/repositories and accession number(s) can be found in the article/Supplementary material.

## Ethics statement

The animal study was reviewed and approved by Committee of the Ethics of Animal Experiments of Kunming Medical University (Permit Number: SCXK 2011–0015). This study conformed to the ethical guidelines of the Science Foundation of National Natural Science of China. Written informed consent was obtained from the individual(s), and minor(s)’ legal guardian/next of kin, for the publication of any potentially identifiable images or data included in this article.

## Author contributions

L-miZ, LC, L-meZ, and M-wL conceptualized and planned the study. LC, Y-fZ, and W-mD performed the experiments. Y-fZ and W-mD analyzed the data. L-miZ wrote the manuscript. All authors have read and approved the final version of the manuscript.

## Funding

This work was supported by the Major Science and Technology Special Project of Yunnan Province under Grant (No. 202102AA100061), Nature Science Foundation of China under Grant (No. 82060252) and (No. 81960350), Yunnan Basic Research Projects under Grant (No. 2018FB115), and the Yunnan Health Training Project of High-level Talents under Grant (No. H-2018058), Yunnan Applied Basic Research Project-Union Foundation of China under Grant (No. 202201AY070001-091), and Applied Basic Research of Yunnan Neurological Disease Diagnosis and Treatment Center under Grant (No. ZX2019-03-05).

## Conflict of interest

The authors declare that the research was conducted in the absence of any commercial or financial relationships that could be construed as a potential conflict of interest.

## Publisher’s note

All claims expressed in this article are solely those of the authors and do not necessarily represent those of their affiliated organizations, or those of the publisher, the editors and the reviewers. Any product that may be evaluated in this article, or claim that may be made by its manufacturer, is not guaranteed or endorsed by the publisher.

## Footnotes

^1^ https://www.ncbi.nlm.nih.gov/gds

^2^ http://www.gsea-msigdb.org/gsea/msigdb/index.jsp

^3^ http://string-db.org/

^4^ http://www.licpathway.net/KnockTF/search.php

^5^ http://mirwalk.umm.uni-heidelberg.de/search_genes/

^6^ https://www.phosphosite.org/homeAction

^7^ www.drugbank.ca

## Abbreviations

TLE: Temporal lobe epilepsy
GEO: Gene expression omnibus
GSEA: Gene Set Enrichment Analysis
SE: Status epilepticus
DEGs: Differentially expressed genes
PTMs: Protein post-translational modifications
qRT-PCR: Quantitative real-time polymerase chain reaction
MsigDB: Molecular signature database
STRING: Search tool for the retrieval of interacting genes
IHC: Immunohistochemistry
MF: Molecular function
CC: Cellular component
BP: Biological process
