# Supplementary figures and images for: Identification of key potassium channel genes of temporal lobe epilepsy by bioinformatics analyses and experimental verification
Source: Front Neurol. 2023 Jul 7;14:1175007. doi: 10.3389/fneur.2023.1175007 (PMC10361730; doi:10.3389/fneur.2023.1175007)

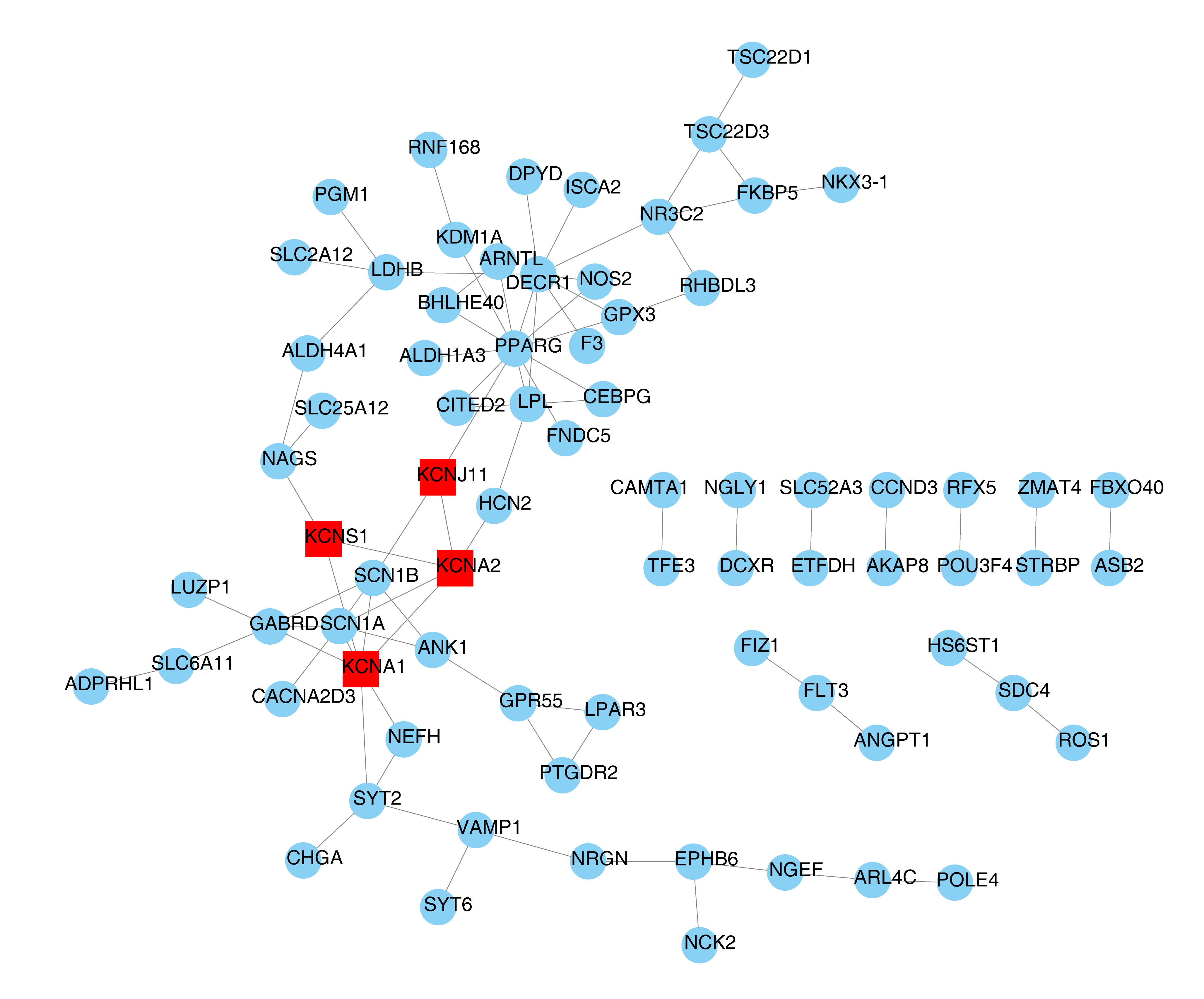

Supplement: Supplementary file 8 [file Image_1.tif]

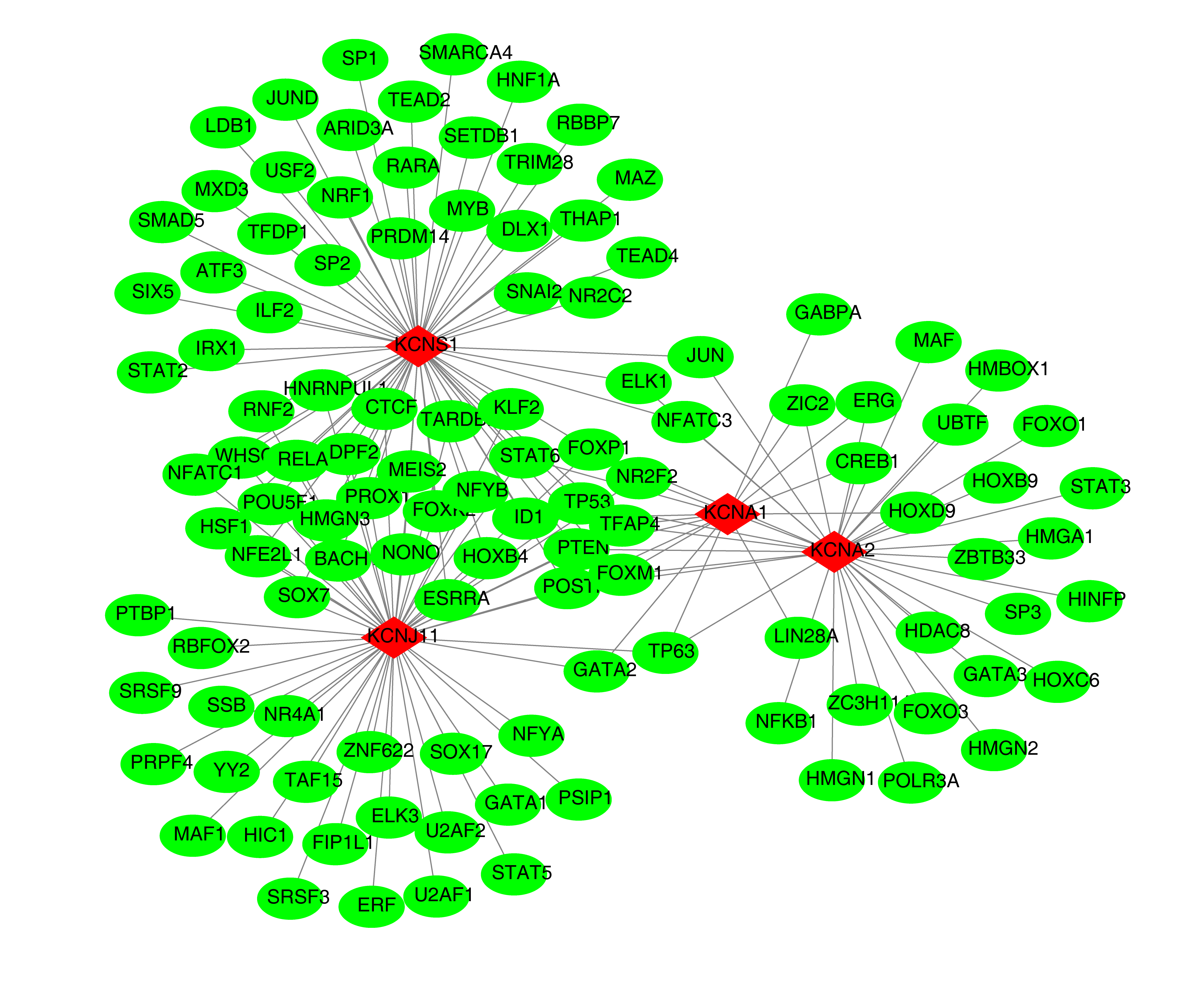

Supplement: Supplementary file 9 [file Image_2.tif]

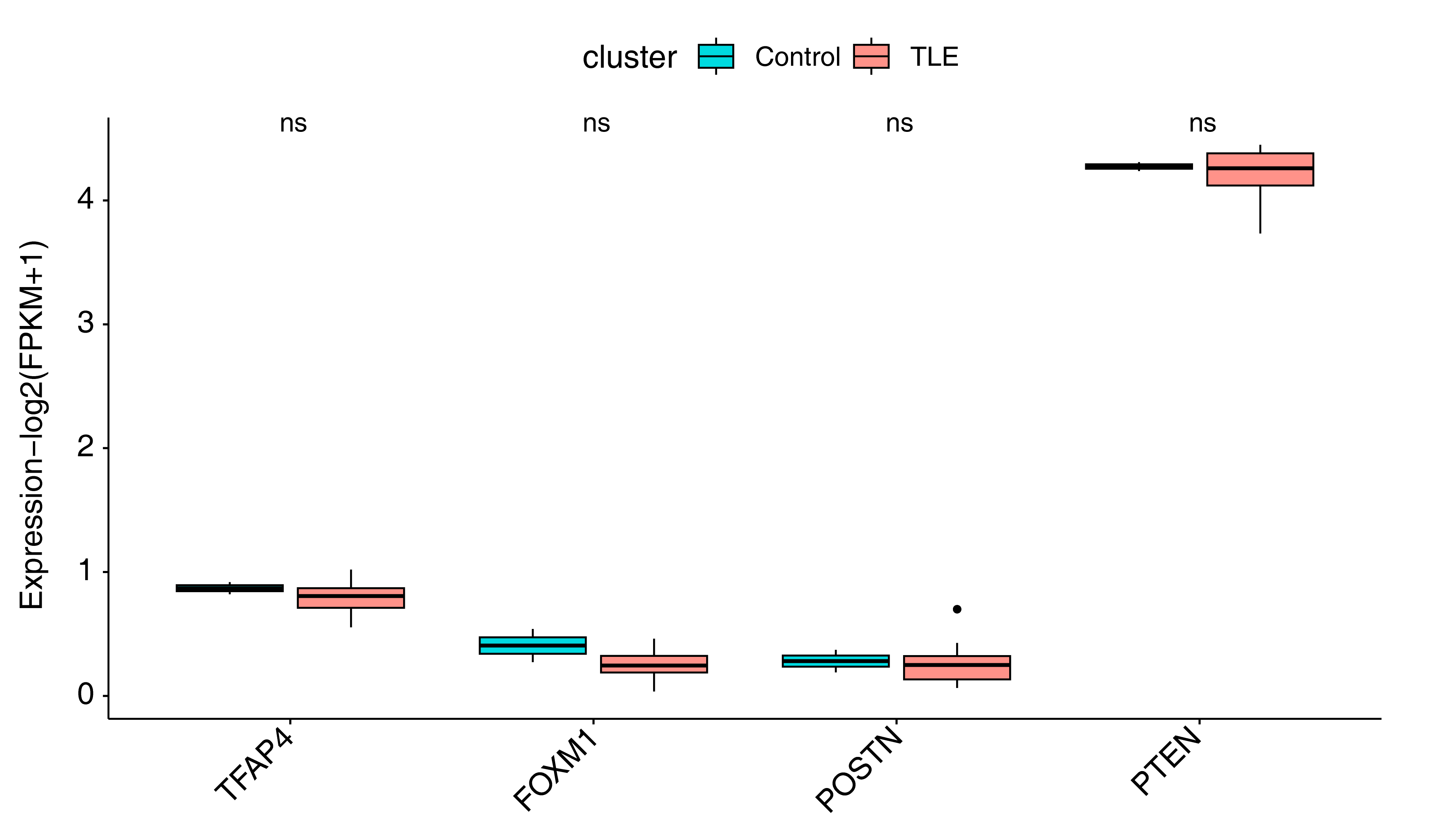

Supplement: Supplementary file 10 [file Image_3.tif]

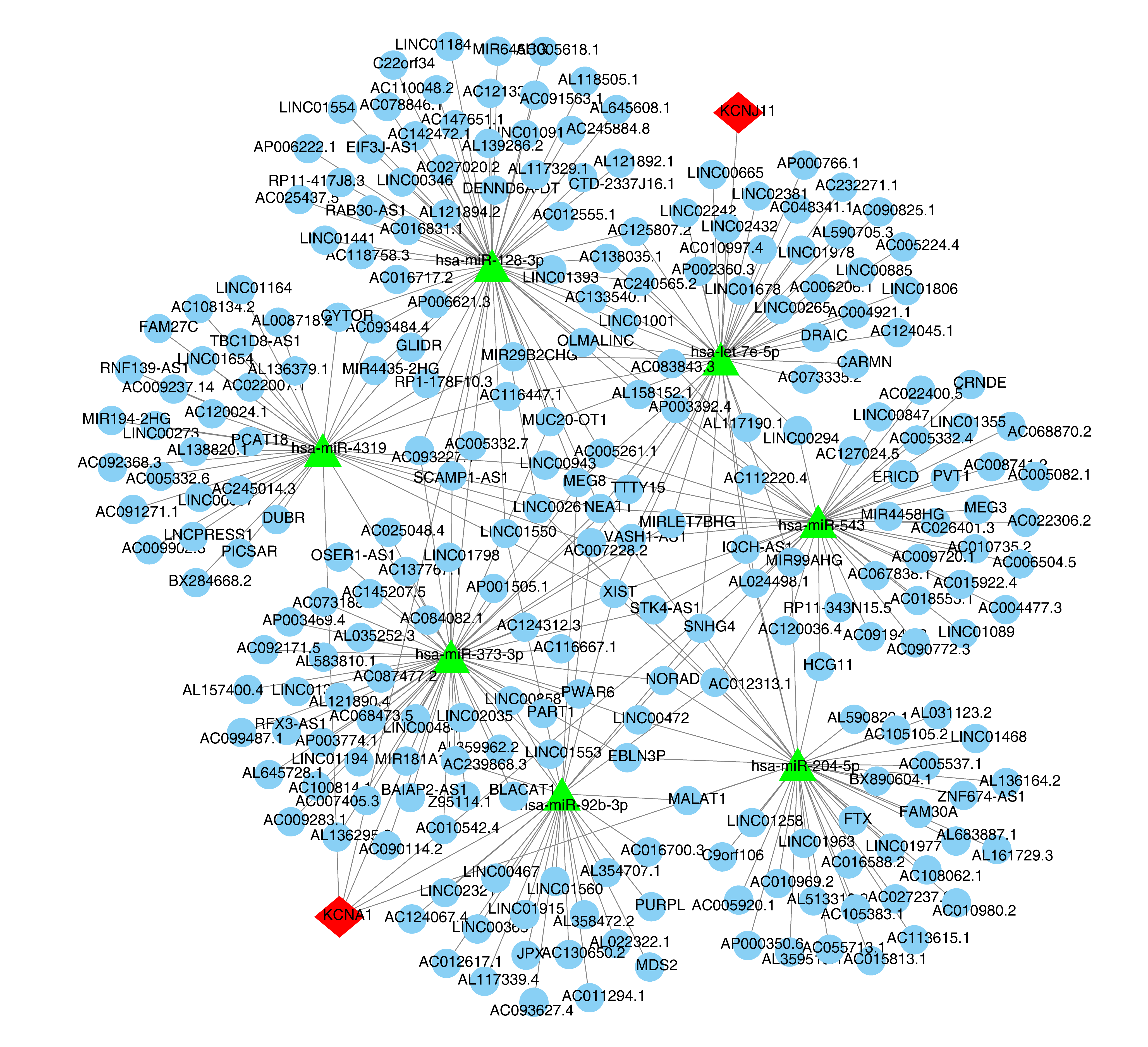

Supplement: Supplementary file 11 [file Image_4.tif]
